# A systematic review of diagnostic tests to detect pelvic floor myofascial pain

**DOI:** 10.1007/s00192-022-05258-7

**Published:** 2022-07-07

**Authors:** Supuni C. Kapurubandara, Basia Lowes, Ursula M. Sansom-Daly, Rebecca Deans, Jason A. Abbott

**Affiliations:** 1 School of Clinical Medicine, UNSW Medicine & Health, Discipline of Obstetrics and Gynaecology, Level 1, Royal Hospital for Women, Barker Street, Randwick, Sydney, NSW 2031 Australia; 2 Department of O&G, Westmead Hospital, Sydney, Australia; 3 Sydney West Advanced Pelvic Surgical Unit, SWAPS, Sydney, Australia; 4 Western Sydney University, Sydney, Australia; 5 Department of O&G, Blacktown Hospital, Sydney, Australia; 6 Behavioural Sciences Unit, Kids Cancer Centre, Sydney Children’s Hospital, Sydney, Australia; 7 Sydney Youth Cancer Service, Nelune Comprehensive Cancer Centre, Prince of Wales Hospital, Sydney, Australia; 8 GRACE Unit, Royal Hospital for Women, Sydney, Australia

**Keywords:** Gynaecological exam, Myofascial pain, Pelvic pain, Pelvic floor myalgia, Persistent pelvic pain, Psychometric

## Abstract

**Introduction and hypothesis:**

Myofascial pain arising from pelvic floor muscles occurs in women with vaginismus, interstitial cystitis and endometriosis but is often overlooked. The aim is to examine alternative diagnostic tests to detect pelvic floor myofascial pain compared with standardized vaginal palpation of pelvic floor muscles as the reference test.

**Methods:**

A systematic review was prospectively conducted (PROSPERO-CRD42020183092) according to PRISMA guidelines. Databases searched included Ovid Medline 1946–, Embase 1957–, Scopus 1960–, Cochrane Combined, Clinical trials, Google Scholar (top 200 articles), Web of Science, TRIP, BIOSIS, DARE, CINHAL, EmCare, PEDro, ProQuest and EBSCOhost up to July 2020. Articles were independently screened by two authors and assessed for bias using QUASDAS-2 tool.

**Results:**

A total of 26,778 articles were screened and 177 were selected for full text review, of which 5 were selected for final analysis. Five studies included 9694 participants of which 1628 had pelvic floor myofascial pain. Only one study reported data to calculate sensitivities and specificities of the index test, which utilized a score of > 40 on the Central Sensitization Inventory to detect women with pelvic floor myofascial pain and revealed a sensitivity of 34.8% and a specificity of 84.9% compared to the reference test.

**Conclusions:**

This systematic review did not reveal any diagnostic test superior to the pre-defined reference test. There is a lack of consensus on the definition of pelvic floor myofascial pain and a lack of a validated diagnostic criteria which must be addressed to progress with meaningful research in this field.

## Introduction

Persistent pelvic pain affects up to one in four women (5.7%–26.6%) [1, 2] and is associated with significant physical, functional and psychosocial impact [3, 4]. Increased cost of living for patients with persistent pelvic pain has been reported to be substantial ($USD 12,406–$USD 15,276 per woman per year) regardless of the cause of pain, largely due to lack of productivity and subsequent economic burden [5]. The extent of pain seems to be strongly associated with increased productivity costs, highlighting the need to optimize pain management in women with persistent pelvic pain [5].

Pelvic floor myofascial pain (PFMP) arising from pelvic floor muscles is a cause of persistent pelvic pain and is associated with changes of urinary, bowel and sexual function [6–8]. Often no single disease entity is found as a cause of persistent pelvic pain, but several contributing conditions can coexist such as vaginismus, interstitial cystitis and endometriosis [9–12]. PFMP is often overlooked in the evaluation, diagnosis and treatment of persistent pelvic pain and therefore its true prevalence is unknown [13]. Estimates of PFMP prevalence therefore range widely from 13%–22% in women with persistent pelvic pain to as high as 78% in women with interstitial cystitis depending on the diagnostic criteria and assessment method utilized [7, 12, 14].

Whilst there is no consensus on diagnostic criteria for PFMP, physical vaginal examination is considered the reference standard test to assess PFMP as it is easy to perform [15, 16] with tenderness on examination considered an uncommon finding in asymptomatic individuals [17]. Vaginal examination of pelvic floor muscles appears reproducible as an assessment tool with good inter- [18–22] and intra-rater [18, 19, 22] reliability being reported.

The aim of this systematic review is to examine alternative diagnostic tests to detect PFMP compared with standardized vaginal palpation of pelvic floor muscles as the reference test.

## Materials and methods

This systematic review was prospectively registered with PROSPERO (CRD42020183092) and conducted according to PRISMA guidelines [23]. To determine the ideal diagnostic test to detect PFMP, this study was also conducted according to both the Synthesizing Evidence from Diagnostic Accuracy TEsts (SEDATE) and the STAndards for Reporting Diagnostic accuracy (STARD) guidelines [24, 25]. Ethics approval was not required because of the study design as a systematic review of the literature.

### Eligibility criteria

The inclusion criteria included any type of study in adult women (> 18 years of age) where the majority (> 50%) of participants were female. Studies included had to incorporate physical vaginal examination to detect pain on palpation of the pelvic floor muscles with another diagnostic method to detect PFMP. The exclusion criteria excluded studies with younger participants (≤ 18 years), studies assessing pelvic pain and pathology occurring in the setting of known iatrogenic complications (i.e., transvaginal mesh), conference abstracts, studies including diagnostic assessment performed as part of measuring outcomes of therapeutic interventions and studies related to pregnancy.

Physical vaginal examination to detect tenderness on palpation of the pelvic floor muscles was the reference test for this systematic review. All other assessment tools were considered index tests. Where multiple diagnostic tests were performed within a study, the most applicable index test to detect pelvic floor myofascial pain was considered and selected by the authors performing the review.

### Search strategy

Search strategies for the diagnosis of PFMP were created with the assistance of an academic medical librarian. Three concepts were implemented for the search strategy: (1) include all types of diagnostic tools, (2) focus on myofascial pain and related disorders and (3) be limited to human studies. The search terms and strategy are detailed in Appendix.

### Information sources

This search strategy was executed in Ovid MEDLINE 1946–2020 (Ovid), Embase 1957–2020 (Ovid), Scopus 1960–2020, Cochrane Combined, Clinical trials, Google Scholar, Web of Science, TRIP, DARE, CINAHL(EBSCOhost), EmCare, PEDro and ProQuest. All searches were from the date of inception of the respective database and completed in July 2020. A hand-search of references from the included studies and relevant reviews took place, and the authors of the primary studies were contacted for clarifications as necessary.

### Study selection

The final search output was screened by two authors independently (S.C.K, B.L.). Screening took place as a two-step process using Covidence systematic review software (Veritas Health Innovation, Melbourne, Australia). Studies that met the inclusion and exclusion criteria and that described any type of assessment test to detect PFMP were included in the abstract screening process to capture any potentially promising assessment tools that have been reported. The full text articles were independently reviewed in detail by the same two authors (S.C.K, B.L.) and included where comparison between two assessment tools was apparent, with physical vaginal examination to detect tenderness on palpation of pelvic floor muscles as the reference test. Studies that did not specifically report sensitivity or specificity but did report data that allowed extraction to construct a 2 × 2 table to assess these measures were included. No restrictions were set with respect to sample size or publication language. Articles published in a language other than English were translated using a web-based translator service. Disagreements in any parameter were discussed with a third author (J.A.A.) to arrive at a consensus.

### Data extraction and assessment of risk of bias

Included studies had data extracted for study design, number of participants, method of recruitment, inclusion and exclusion criteria, index or modelled index test, reference or modelled reference test, blinding, population description, primary and secondary outcome results, sensitivity, specificity, limitations and concluding findings. Modelling of an index or reference test was useful in studies that included several possible diagnostic tools, whether comparatively or as part of a broader clinical assessment. Data extraction was performed independently by two authors (S.C.K., B.L.), and any disagreements were resolved with discussion with the third author (J.A.A.) to arrive at a consensus.

Risk of bias and applicability of each study were analyzed using the Quality Assessment of Diagnostic Accuracy Studies (QUADAS-2) tool [26]. Quality assessment was not performed as per the guidance for reviews involving diagnostic test analyses [27]. The risk of bias was described in relation to four domains including patient selection, index test, reference standard and flow and timing [26]. This assessment was performed independently by two authors (S.C.K., B.L.) and any disagreements were resolved by discussion with the third independent author (J.A.A.) to arrive at a consensus.

### Data synthesis

The primary outcome measure was the sensitivity and specificity of the index test compared with physical vaginal muscle examination to detect PFMP. The data are presented in a narrative form in the absence of sufficient data to perform a quantitative synthesis.

## Results

### Study selection

**Fig. 1.**
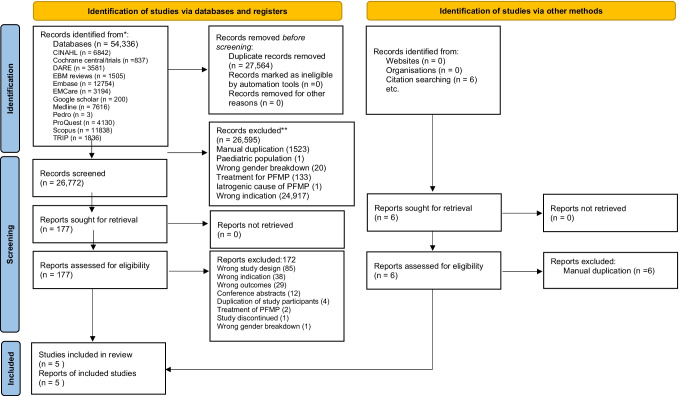
Preferred Reporting Items for Systematic Reviews and Meta-Analyses (PRISMA flow) diagram for systematic review

A total of 26,778 articles were screened with 177 selected for full text review from which five studies [22, 28–31] were selected for final review (Fig. 1). Only one of the five studies allowed the determination of the sensitivity and specificity of the index test (Table 1) [30]. Following full text review, 48% (85/177) of studies were excluded as they used a methodology or described results that did not allow for data extraction to determine diagnostic test accuracy. This included 26% (22/85) of studies where only the prevalence of symptoms or signs was reported.

**Table 1.** Study characteristics of included studies

Author, year, country	Study design	Setting	Sample size	Cases with PFMP	Age of population	Index test	Reference test
Vandyken, 2020, Canada	Nested prospective case control	Multicenter	99	33	Mean 40.6 (± 12.7)	Central sensitization inventory (CSI)	Pain with palpation of pelvic floor muscles (levator ani, obturator internus and iliococcygeus) (dichotomous)
Aw, 2017, Australia	Prospective cohort	Single center	201 (193 women)	85 (84 women)	Median age 51 vs 59 years (asymptomatic)	Cystoscopy, profilometry, Mid-urethral closure pressure (MUCP)	Tenderness with palpation of pelvic floor muscles (dichotomous)
Bhide, 2015, Canada and UK	Prospective case control	Multicenter	111	44	Mean 40 vs 60 (asymptomatic)	Pelvic floor muscle hyperalgesia (PFMH) score (0–III)	Pain with palpation of pelvic floor muscles (levator ani, piriformis and internal obturator) (VAS score 0–10)
Droz, 2011, USA	Retrospective cohort	Single center	331	33	Median age 34 (15–82)	McGill Pain Questionnaire pain descriptor	Moderate or severe tenderness of levator ani
Adams, 2013, USA	Retrospective cross-sectional	Multicenter	8960	1434	Mean age 56.8 years vs 65.5 (WO LM)	PFDI, PFIQ	Pain with palpation of pelvic floor muscles (pubococcygeus, iliococcygeus and obturator internus) (dichotomous)

VAS = visual analogue scale

PFMP = pelvic floor myofascial pain

PFDI = Pelvic Floor Disability Index

PFIQ = Pelvic Floor Impact Questionnaire

### Study characteristics

The study characteristics of the five included studies are summarized in Table 1.

Three of the five studies recruited patients prospectively (1 cohort, 2 case-control studies) [22, 30, 31]. The retrospective study with the largest cohort of women (8960 women with 1434 with PFMP) only included 88 women with PFMP compared with 88 women without PFMP in the final analysis due to significant missing data (2229 pelvic examination findings) [28]. Only three of the five studies mentioned the objective of finding a diagnostic test but were not designed in a way that allowed assessment of diagnostic test accuracy [22, 29, 31]. The diagnostic tests utilized in the five studies included the Central Sensitization Inventory [30], questionnaires (Pelvic Floor Disability Index [28], Pelvic Floor Impact Questionnaire [28] and McGill Pain Questionnaire [29]), pelvic floor muscle hyperalgesia (PFMH) scoring system [22] and urodynamic parameters [31].

The inclusion criteria were mentioned for all studies; however, the exclusion criteria were not mentioned in one study [31]. The time points of patient recruitment were not mentioned in one study [22]. Two studies included both men and women; however, the outcomes of one study were based predominantly on findings in women (96%) [31] and the other study excluded men from the final analysis because of small numbers of men recruited (*n* = 3) [30].

The assessors were not blinded to the patients’ clinical presentation or to the outcomes of the index test and reference test in four of the studies [22, 28, 29, 31]. The timeline between the administration of the index and reference tests was uncertain in all studies [22, 28–31]. All index tests were described in a way that allowed for replication of the test. The reference test was only described in a way to allow replication in three studies [22, 30, 31] with none of the studies describing standardization of pressure on physical examination with an algometer. Two studies did not mention how many assessors were used [28, 29] with the remaining studies using one [31], two [22] and four [30] assessors, respectively. Only two studies mentioned the experience of the assessors conducting the examination [30, 31].

Only one study reported raw data in a way that allowed the calculation of the sensitivity and specificity for the index test [30]. Inter- and intra-rater reliability was calculated and available in one study [22].

### Risk of bias of included studies

**Fig. 2.**
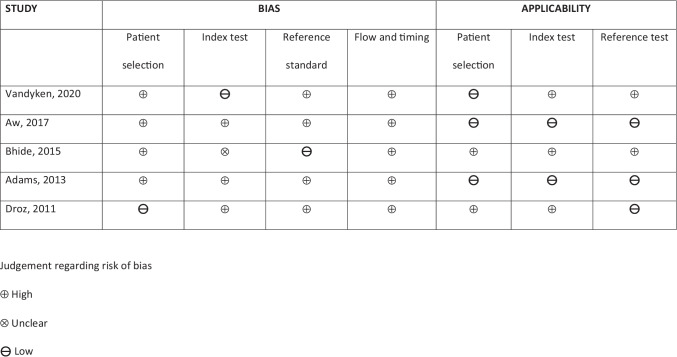
Traffic-light plot summarizing the authors' review of the Quality Assessment of Diagnostic Accuracy Studies (QUADAS-2) risk of bias and applicability concerns

**Fig. 3.**
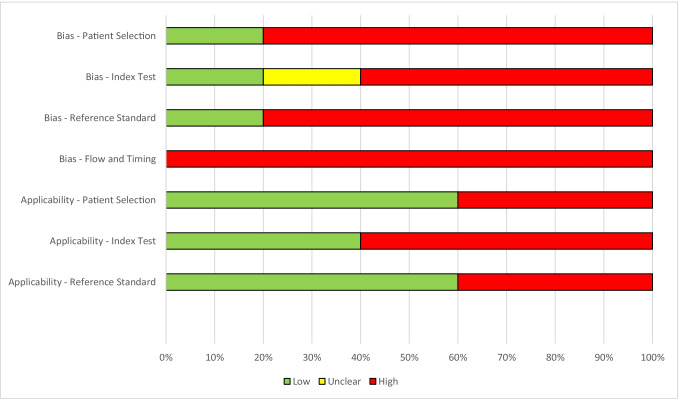
Quality Assessment of Diagnostic Accuracy Studies (QUADAS - 2) tool to quality evaluation of all five included studies

Risk of bias of the included studies is summarized in Figs. 2 and 3.

All five studies had high risk of bias for flow and timing of both index and reference tests [22, 29, 30]. The index test was only applicable in three studies [22, 29, 30] and the reference test was highly applicable to the clinical population of interest in only two studies [22, 30].

### Synthesis of results

Only one study, which compared a score of > 40 on the Central Sensitization Inventory to the reference test, reported data in a way to calculate a sensitivity of 34.8% and a specificity of 84.9% to detect PFMP with a false-negative rate of 65% and a false-positive rate of 15% [30]. The remaining four studies met the inclusion and exclusion criteria but did not have enough information to determine sensitivity and specificity of the index test.

Only one study reported reproducibility with an intra-observer reliability of ICC 0.43–0.80 (moderate to excellent) and an inter-observer reliability of ICC 0.72–0.91 (good to excellent) [22].

## Discussion

### Principal findings

This is the first systematic review to determine the ideal alternative diagnostic test to detect PFMP using tenderness on vaginal pelvic floor examination as a reference test. This review represents the most rigorous examination of diagnostic tests to detect PFMP to date, examining international literature spanning multiple databases since their respective inceptions over 50 years. Despite this rigor, no studies formally designed to assess diagnostic test accuracy specific to PFMP were found, with only five studies ultimately eligible for inclusion highlighting a considerable gap in diagnostic research pertaining to PFMP. Therefore, conclusions about the ideal diagnostic test cannot be drawn at this time because of insufficient evidence.

This review suggests that standardized physical vaginal examination of pelvic floor muscles to detect tenderness be utilized to detect PFMP with the addition of an algometer or pressure device not required to improve diagnostic capability. Physical examination to detect tenderness appears most practical [15, 16] with good reported inter- [18–22] and intra-rater [18, 19, 22] reliability. PFMP may occur either as an isolated diagnosis or in conjunction with other conditions including musculoskeletal [32, 33], gastrointestinal [34–36], genitourinary [31, 37–39], gynaecological [6, 40, 41] and persistent pelvic pain [20, 42] conditions. PFMP may present as primary muscular dysfunction [43, 44] or as a consequence of peripheral and central sensitization resulting from other pain conditions, as reflected in studies demonstrating extra pelvic manifestations in women with pelvic pain [6, 45–49], mucosal hypersensitivity [50], enhanced pain sensitivity [51–54], regional allodynia and hyperalgesia [6]. Assessing for peripheral and central pain mechanisms contributing to PFMP is important to tailor management, which may require multimodal interventions [55] including physical and medical therapy, such as botulinum toxin [56], rather than physical therapy alone [57].

Of the five studies included in the analysis, only one had data sufficiently reported in a way to derive sensitivity and specificity with Central Sensitization Inventory as the index test, which was less effective compared to vaginal examination to detect PFMP [30]. Further deductions cannot be made from this study given the nested case-control design, inability to calculate accurate positive and negative predictive values, small numbers and high risk of bias in most domains described [30].

Of the remaining studies, two [28, 29] looked at the utility of questionnaires (Pelvic Floor Disability Index, Pelvic Floor Impact Questionnaire and McGill Pain Questionnaire) with the reference test. The third study reviewed a novel pelvic floor scoring system [pelvic floor muscle hyperalgesia(PFMH) score] with the reference test which in itself demonstrated good intra-observer (ICC = 0.43–0.80) and inter-observer reliability (ICC = 0.72–0.92) [22], consistent with other studies described in the medical literature [18–21]. The fourth study demonstrated altered urodynamic parameters in women refractory to conservative management of lower urinary tract symptoms, with a higher mid-urethral closure pressure (93.1 cmH_2_O vs. 80.6 cmH_2_O, *p* = 0.015) noted in women with PFMP [31]. Overall, the remaining four studies had methodological flaws and concerns regarding risk of bias and their applicability, making it difficult to draw any further conclusions.

### Comparison with existing literature

Many different types of questionnaires are used to assess PFMP including Pelvic Floor Distress Inventory [7, 8, 28], Pelvic Floor Impact Questionnaire [7, 28], Pelvic Pain, Urgency and Frequency [7], Central Sensitization Inventory [30] and McGill’s pain questionnaire [29]. With no questionnaire specific to PFMP, there is scope for future development of such a questionnaire as the symptom profile of women with PFMP is determined.

Several diagnostic tests identified in this review require further study to specifically detect PFMP. The modified Oxford Scale has been described to objectively assess tone [58], which is only comparable to manometry in patients with suspected reduced tone [59, 60]. Standardized pelvic floor muscle assessment techniques incorporating tone as a component of the overall assessment has also been described, for example, the PERFECT (pressure, endurance, repetitions, fast contractions, every contraction timed) scheme, but none of these tools have been assessed specifically to detect PFMP [61]. Dynamometry has been utilized to assess resistance, endurance and strength of pelvic floor muscles [62, 63] and has demonstrated overall good reliability [64] and diagnostic accuracy compared to the modified Oxford Scale [65]. The utility of this assessment may also be limited by lack of access to the device outside a research setting and the limited expertise of clinicians in its use. Vaginal manometry is another objective way to assess muscle pressure [59, 60] and demonstrate good inter-rater reliability compared to digital examination [60]. Whilst easily accessible, further evidence is required to determine the applicability in women with PFMP.

Electromyography (EMG) has been used to distinguish neural drive to muscles [66–68] but limitations include access to equipment, expertise in its use, lack of an appropriate vaginal probe and the probability for artefact and crosstalk from other muscles [68]. In one case-control study, turn-amplitude analysis by a single operator (using EMG) seemed to be a promising diagnostic test to detect PFMP compared to clinical judgment incorporating pelvic floor examination as a comparator (*n* = 128). This study was excluded from the final analysis as it did not have > 50% of women (*n* = 64) within the study participants [69]. This study reported the test having a sensitivity of 83%, specificity of 100%, positive predictive value of 1 and negative predicted value of 0.85; however, further research is required to demonstrate external validation and reproducibility [69].

Ultrasound (both trans-perineal and trans-vaginal) [35, 70, 71] and magnetic resonance imaging (MRI) [72] are emerging imaging modalities for assessment of pelvic floor muscle morphometry. Ultrasound seems easily accessible in contrast to MRI; however, both imaging modalities are limited by access to experienced clinicians able to reliably report findings. Whilst a smaller study [71] suggested changes in muscle morphometry on ultrasound in patients with potential pelvic pain, a larger prospective study [70] reviewing 368 nulliparous women did not demonstrate any difference in muscle morphometry in the presence of pelvic pain. It is also important to note that multiple variables can influence muscle morphometry including parity, age [73], pelvic floor trauma [74] and ethnicity [75], which requires normative data from large cohorts to understand the utility of such imaging tests to detect PFMP and whether there is any correlation between structure of muscles from their function.

### Strengths and limitations

The strengths of this systematic review include the broad search terms, a robust methodology and the adoption of PRISMA guidelines to perform this review [23]. Descriptive studies that described an assessment test to detect PFMP without a comparator were specifically excluded. This review revealed a number of diagnostic tests for assessing PFMP including dynamometer [66], surface electromyography [66, 76], algometry [53], morphometry [77, 78], manometry [76] and questionnaires [7, 8, 22]. A recent systematic review of physical examination techniques to detect PFMP included 55 studies, however, only two assessed the examination itself, with most included studies being clinical reviews and prevalence studies [15]. In contrast, our review only reviewed studies designed to assess diagnostic capabilities to detect PFMP compared with physical examination. The findings of this review highlight that whilst there are various related publications on the topic of PFMP, they are heterogenous with respect to the definition of PFMP utilized, study design and study objectives [15].

The limitations of this review include the narrow inclusion and exclusion criteria, which were essential in determining the most appropriate diagnostic test to detect PFMP. Given the paucity of evidence in this area, exclusions were not made based on study design or the number of study participants, which could lead to the inclusion of low-quality and underpowered studies in this review. Exclusion of studies examining the detection of PFMP in association with conditions other than gynaecological presentations are not captured in this review [32, 34, 35]. Future research and development of reliable diagnostic tests for PFMP need to consider the assessment of women who may not tolerate an invasive clinical examination, including women with acute pain, difficulties with vaginal penetration and who demonstrate signs of central sensitization who may experience worse symptoms with repeat examinations [54, 79]. This systematic review highlights the extremely limited data regarding patients’ perspectives and experience with PFMP. Incorporating such information is essential in this area of persistent pain diagnosis and management that will improve quality of life for women with this condition.

### Conclusions and implications

This review suggests that a standardized physical vaginal examination of pelvic floor muscles to detect tenderness offers good inter- and intra-rater reliability and should be utilized to detect PFMP until further advancements are made in diagnostic research of this clinical condition [16, 18–22, 42]. This is with a caveat of limited knowledge on the aetiology of PFMP [44, 80] and a lack of uniformity in the diagnostic criteria and definition used in this diagnosis. These important parameters must be first addressed before further meaningful research in this field can progress. Future studies assessing diagnostic tests to detect PFMP should be conducted in a way to assess diagnostic test accuracy to determine the ideal diagnostic tool.

## Appendix

### Search Strategy: Medline

Three main concepts were implemented into the search strategy including all types of diagnostic tools and myofascial pain and related disorders, limiting to studies pertaining to humans.

The terms for diagnostic tools included: “psychometric”, “psychometrics”, “diagnostic test, routine/”, “surveys and questionnaires”, “pelvic floor muscle assessment”, “ pelvic muscle assessment”, “pelvic floor muscle exam”, “gynaecological exam”, “gynecological exam”, “transvaginal pelvic floor exam”, “pelvic exam”, “screening exam”, “internal exam”, “gynaecological examination”, “palpation”, “turns-amplitude analysis”, “physical examination”, “diagnostic imaging”, “muscle strength dynamometer”, “dynamometer”, “perineometer”, “electromyography”, “algometer”, “pain measurement”, “routine diagnostic test”, “musculoskeletal screening”, “vaginal exam”, “diagnos*”, “valid”, “scale”, “reliable test”. The terms for myofascial pain and related disorders included “myofascial pain syndrome”, “pelvic floor disorders”, “pelvic floor”, “myofascial syndrome”, “myofascial pain”, “chronic pelvic pain”, “persistent pelvic pain”, “pelvic pain syndrome”, “pelvic pain”, “pain”, “syndrome”, “dysfunction”, “ache”, “spasm”, “myalgia”, “soreness”, “tenderness”, “trigger point”, “viscerosomatic reflex”, “somatovisceral convergence”, “levator ani”, “vaginismus”, “dyschesia”, “dyschesia”, “non menstrual pelvic pain”, “levator myalgia”, “dysmenorrhea”, “vaginal pain”, “painful defecation”, “dyspareunia”, “high tone pelvic floor dysfunction”, “painful sex”.
